# *Dendrobium candidum* polysaccharide reduce atopic dermatitis symptoms and modulate gut microbiota in DNFB-induced AD-like mice

**DOI:** 10.3389/fphys.2022.976421

**Published:** 2022-09-09

**Authors:** Yiheng Liang, Guangrong Liu, Lingna Xie, Kewen Su, Xia Chang, Yani Xu, Junsong Chen, Zhenyuan Zhu, Kaiye Yang, Huixiong Chen, Zhiyun Du

**Affiliations:** ^1^ School of Biomedical and Pharmaceutical Sciences, Guangdong University of Technology, Guangzhou, China; ^2^ Guangdong Provincial Key Laboratory of Plant Resources Biorefinery, Guangdong University of Technology, Guangzhou, China; ^3^ Research and Development Center, Infinitus (China) Co., Ltd., Guangzhou, China; ^4^ State Key Laboratory of Food Nutrition and Safety, College of Food Science and Engineering, Tianjin University of Science and Technology, Tianjin, China; ^5^ Chemistry of RNA, Nucleosides, Peptides and Heterocycles, CNRS UMR8601, Université Paris Descartes, PRES Sorbonne Paris Cité, UFR Biomédicale, Paris, France

**Keywords:** atopic dermatitis, gut microbiota, plant polysaccharide, inflammatory cytokine, dinitrofluorobenzene

## Abstract

Atopic dermatitis (AD) is a chronic inflammatory skin disease with a high prevalence worldwide. Increasing evidence suggests that the gut microbiota plays an important role in the pathogenesis of AD. In this study, we sought to verify the effect of *Dendrobium candidum* polysaccharides (DCP) on AD induced by 2,4-Dinitrofluorobenzene (DNFB) in Balb/c mice regarding its impact on the intestinal microbiome. We found that 2-week oral administration of DCP improved AD-like symptoms and histological damage of skin, reduced mast cell infiltration, down-regulated the level of serum total IgE and the expression of pro-inflammatory cytokines such as TNF-α, IFN-γ, IL-4 and IL-6, and increased the expression level of anti-inflammatory cytokine IL-10. The beneficial effect of DCP was attributed to the restoration of the intestinal microbiome composition and the unbalance of the intestinal homeostasis. Our results indicated that DCP might be used as a promising novel microbiota-modulating agent for the treatment of AD.

## Introduction

Atopic dermatitis (AD), often referred to as eczema, is a chronic disease that causes inflammation, redness, rashes and irritation of the skin. AD typically begins in childhood, usually in the first 6 months of a baby’s life. Some children who develop AD may continue to have symptoms as teens and adults. In people with AD, the immune system becomes disordered and overactive, which triggers inflammation and damages the skin barrier, leaving it dry and prone to itching and rashes (Torres et al., 2019). No one knows what causes AD, but there are many contributing factors such as genes, the immune system, microbiota dysbiosis and skin barrier dysfunction which play a key role in the disease (Sroka-Tomaszewska and Trzeciak, 2021).

Local corticosteroids (TCSS) and calcineurin inhibitors (TCI) are two primary families of agents currently used to treat AD. Although these therapies can be helpful, prolonged application of TCSS results in the skin atrophy and other local complications including striae, hypopigmentation, telangiectasia and acne (Pariser, 2009). There is also concern that TCI can cause systemic immunosuppression and has a possible link to lymphomas and skin malignancies (Siegfried et al., 2016; Arana et al., 2021). Therefore, it is particularly important to develop drugs that have limited harmful side effects for AD patients.

*Dendrobium candidum* (DC) has been widely used in traditional Chinese herbal medicine in many Asian countries (Lam et al., 2015). There are more than 60 species of Dendrobium which have been found in China. Many studies have shown that DC had beneficial effects on antioxidant, immunostimulatory and antitumor activities and dermatological disorders (Moretti et al., 2013; Guo et al., 2019; Zhang et al., 2019; Wang, 2021). DC polysaccharides (DCP) is one of the principal active ingredients in DC, which can regulate the oxidative stress and the proliferation of human umbilical vein endothelial cells, has strong antioxidant and anti-hyperglycemic properties, and is effective in promoting hair growth and in skin hydration (Zhao et al., 2007; Chen et al., 2014; Cha et al., 2021). Recent study also showed that DC polyphenols can alleviate zebrafish intestinal inflammation by regulating the intestinal microbiota (Gong et al., 2020).

It is well known that gut microbiota and their repertoire of biochemical reactions contribute to many aspects of host health, including metabolism, immunity, development, and behavior (Kim et al., 2013; Janssen and Kersten, 2017; Gebrayel et al., 2022). Microbial dysbiosis, which is an imbalance of the microbial community, can contribute to the development of numerous diseases (Zuo and Ng, 2018; Kim et al., 2020; Liu et al., 2020). With the development of sequencing technologies, many studies demonstrate that AD is closely linked to changes in the diversity and composition of the gut microbiota, especially in early life (Lee et al., 2018b). The gut microbiota is involved in the metabolism of short-chain fatty acids (SCFA), amino acids, vitamins and bile acids to induce maturation of the innate and adaptive immune system (O’Hara and Shanahan, 2006). The dysbiosis of the gut microbiome combined with the immune system imbalance contributes to the development of AD. Currently, microbial-based and microbial-targeted therapies are emerging strategies for inflammatory bowel disease to show favorable results (Oka and Sartor, 2020). Although the mechanisms of crosstalk between the gut microbiota and the skin disease remain to be explored, the current concept of the “gut-skin” axis has been recognized as a new target for the prevention and treatment of AD (Park et al., 2021).

Thus, we speculated that DCP might regulate gut microbiota, modulate host immune system and reduce AD symptoms. In this study, DCP were prepared and characterized by monosaccharide compositions, Fourier transform infrared spectroscopy (FT-IR) and nuclear magnetic resonance (NMR). The effects of DCP on LPS-stimulated RAW 264.7 cells and their impacts on the improvement of the symptoms of AD in 2,4-Dinitrofluorobenzene (DNFB)-induced mice were also investigated. This study provides insight into the effects of DCP on the gut microbiota and new evidence for their impacts on AD.

## Materials and methods

### RAW264.7 cell viability

RAW264.7 cells were cultured in DMEM cell culture medium (containing 10% FBS, 1% penicillin) in an incubator at 37°C and 5% CO_2_. After grown to about 80%, the cells were exchanged and passaged. The cell algebra used in the experiment was 2–10 generations. 100 μl of RAW264.7 cell suspension was added to a 96-well plate (1 × 10^4^ cells per well) and incubated in a 37°C incubator for 24 h. The medium was discarded and 0.02% (20 μg/ml), 0.1% (100 μg/ml) and 0.5% (500 μg/ml) of DCP in the same culture medium were added. After 24 h of incubation in a 37°C incubator, 0.5 mg/ml MTT was added to each well and incubated in a 37°C incubator for 4 h (shading treatment). The solution was decanted and 150 μl DMSO was added to each well for 15 min. The OD value was measured at 570 nm using a microplate reader for 10 min. The experiment was set up with six replicate wells and repeated three times.

### Nitric oxide and cytokine measurements

2 ml of RAW 264.7 cell suspension was added to a 6-well plate (1 × 10^5^ cells per well) and incubated in a 37°C incubator for 24 h. The medium was discarded and 2 ml of 5 μg/ml LPS solution was added. After 2 h of incubation in a 37°C incubator, 0.02%, 0.1%, 0.5% DCP was added to each well and cultured in a 37°C incubator for 24 h 50 μl of the supernatant was aspirated into 96 plates, and the Nitric oxide (NO) content in the collected culture supernatant was determined by the Griess method (Dong et al., 2018). The supernatant was mixed with the Griess reagent in equal volume, and after 5 min of reaction. The OD value was measured at 540 nm using a microplate reader for 10 min. The production of the pro-cytokines (TNF-α and IL-1β) was measured using an enzyme-linked immunosorbent assay (ELISA) kit (Jiangsu Meibiao Biological Technology Co., Ltd.) according to the manufacturer’s protocol. This cell experiment was set up with six replicate wells and repeated three times.

### Ethics statement

All experiments were conducted following relevant laws and regulations and approved by the Laboratory Animal Center of Wuyi University (SCXK/2016-0041). All efforts were made to minimize animal suffering.

### Animals and experimental design

24 female BALB/c mice (6-week-old) were purchased from the Animal Experimental Center of Sun Yat-sen University. BALB/c mice were fed and libitum for 1 week before the experiment. They were placed in an air-conditioned animal room with 12 h of light and dark lighting and constant humidity. Under the conditions of a temperature of 22 ± 1°C and a humidity of 50% ± 10%, the mice were provided with a normal diet and drinking water.

**FIGURE 1:**
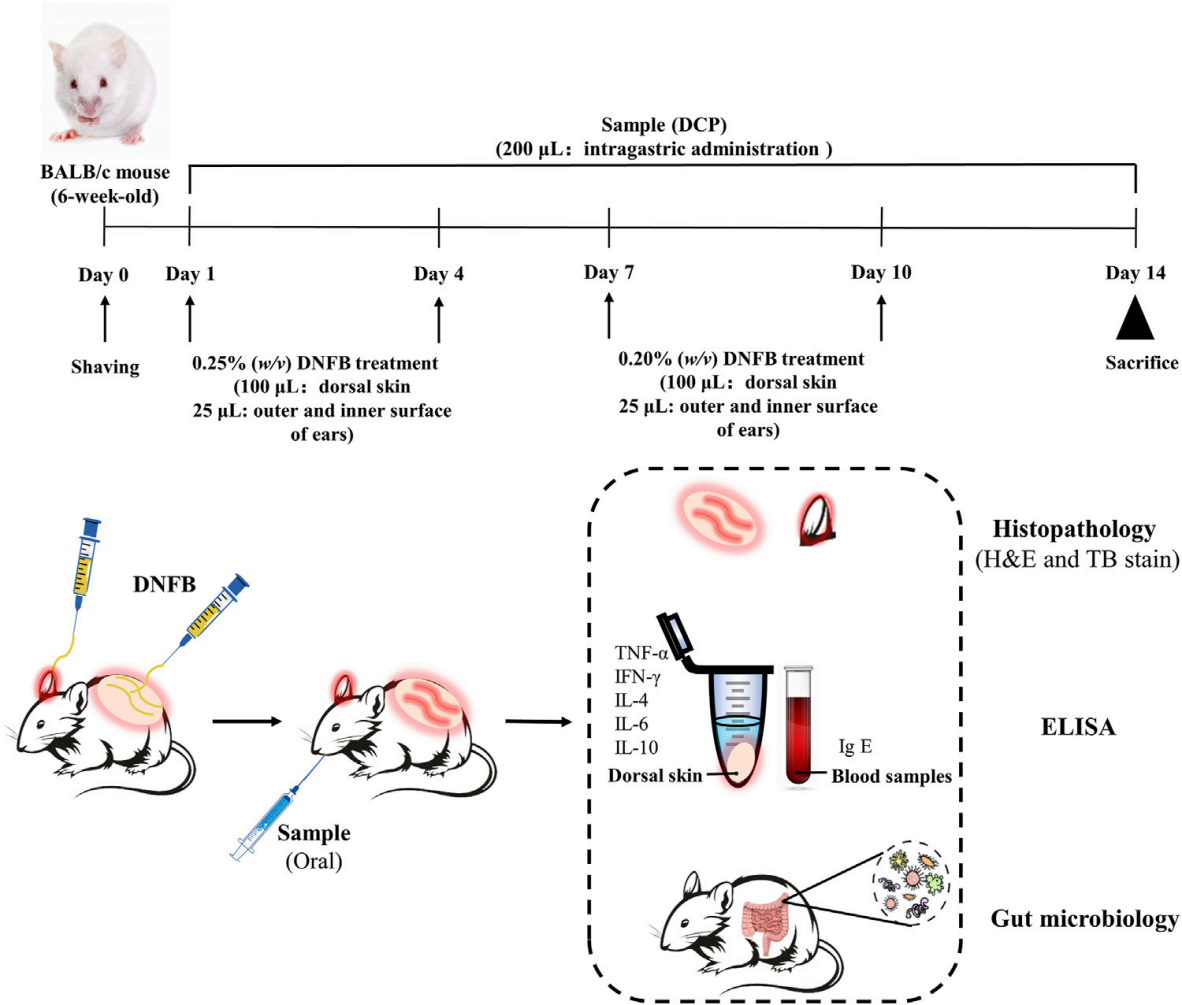
Experimental protocol for DNFB-induced AD-like lesions and oral treatment of DCP in BALB/c mice. 24 female BALB/c mice (6-week-old) were randomly divided into four groups (*n* = 6 per group): Blank control group, DNFB-induced AD-like model group, treatment group with DCP at 50 mg/kg and treatment group with DCP at 200 mg/kg.

The experimental scheme was summarized in Figure 1. The BALB/c mice were randomly divided into four groups. They were: Blank control group, DNFB-induced AD-like model group, Treatment group with DCP at 50 mg/kg (DNFB + DCP-L), Treatment group with DCP at 200 mg/kg (DNFB + DCP-H). First and fourth days after shearing the dorsal skin of mice, 100 and 25 μl of 0.25% (*w/v*) DNFB solution in acetone-olive oil (3:1) was applied to the dorsal skin and ears. On day 7 and day 10, the dorsal skin and ears were stimulated with 100 and 25 μl of 0.2% (*w/v*) DNFB respectively. DCP was dissolved in sterilized drinking water, then 0.2 ml of 50 or 200 mg/kg DCP was administered daily by gavage to the treatment groups. On day 14, mice were anaesthetized using intraperitoneal injection of sodium pentobarbital (1%, 50 mg/kg). Blood samples were collected from the eyelids by retro-orbital bleeding (ROB), centrifuged (12,000 rpm, 10 min) and stored at −80°C. Subsequently, the animals were sacrificed by decapitation, dorsal skins and ears were collected and stored at −80°C. 6 mm diameter ear biopsies of each group were obtained using a hole puncher and weighted. Fecal samples were collected from the colon immediately and stored at −80°C for further gut microbiota analysis.

### Skin evaluation and histological assessment

Based on the severity of erythema/hemorrhage, erosion, dryness and scabbing, the degree of skin damage was assessed by five independent investigators in a blinded randomized analysis to limit investigator bias and variability, and categorized as 0 (none), 1 (mild), 2 (moderate) and 3 (severe), according to the method previously described (Kim et al., 2013). The total SCORAD (mean ± SD, minimum 0, maximum 12) was used as the total score for each mouse.

For the histological assessment, the dorsal skin and ear samples of BALB/c mice were removed, fixed in 4% paraformaldehyde and embedded in paraffin. The sections were subjected to HE staining with hematoxylin and eosin solution and to toluidine blue staining with toluidine blue working solution (1% toluidine blue and 1% aluminum potassium sulfate). The slides were imaged using a Nikon optical microscope (Japan) equipped with an eyepiece micrometer by the magnification (×200) and analyzed using Zen 2.3 SP1 software (Carl Zeiss). Mast cells are labeled and counted using image pro plus 6.0 (Measure/count/size plug-in).

### Enzyme-linked immunosorbent assay

Serum total IgE levels were determined using an ELISA kit (Jiangsu Meibiao Biological Technology Co., Ltd.) according to the manufacturer’s instructions.

20 mg of dorsal skin tissues from BALB/c mice was homogenized in 200 μl RIPA Lysis Buffer (Guangzhou Dingguo Biological Technology Co., Ltd.) containing 1% protease inhibitor cocktail (Guangzhou Dingguo Biological Technology Co., Ltd.). The supernatant was collected by centrifugation at 12,000 rpm for 10 min at 4°C and then tested with ELISA kits (Jiangsu Meibiao Biological Technology Co., Ltd.) to determine the levels of TNF-α, IFN-γ, IL-4, IL-6 and IL-10 according to the manufacturer’s instructions.

### Gut microbiota analysis

0Gut microbiota profiling of fecal samples from blank control, DNFB-induced AD-like model and 200 mg/kg DCP treatment groups were performed by Shanghai Majorbio Bio-Pharm Technology Co., Ltd. Microbial DNA was extracted from fecal samples by using the TIANamp Stool DNA Kit (TIANGEN). The V3-V4 regions of the bacteria 16S ribosomal RNA gene were amplified by PCR using the flowing primers: 338F 5′-ACT​CCT​ACG​GGA​GGC​AGC​AG-3′, 806R 5′-GGACTACHVGGGTWTCTAAT-3′. The PCR products were then extracted from 2% agarose gels for further purification and quantification.

The purified DNA amplicons were added with Illumina adapters by ligation (TruSeq DNA LT Sample Prep Kit), and the adapter-ligated DNA fragments were further pooled in equimolar and paired-end sequenced (2 × 300) on an Illumina MiSeq platform for sequencing according to the standard protocols by Majorbio Bio-Pharm Technology Co. Ltd. (Shanghai, China). The DNA library is multiplexed and loaded onto the Illumina MiSeq instrument, according to the manufacturer’s instructions (Illumina, San Diego, CA, United States). The data were analyzed on the online platform of Majorbio Cloud Platform (www.majorbio.com). To obtain the species classification information corresponding to each OTU, the RDP classifier Bayesian algorithm was used to carry out taxonomic analysis on the representative sequences of OTUs at each taxonomic rank (Domain, kingdom, Phylum, Class, Order, Family, Genus, Species) with 97% similar level and to count the community species composition of each sample. Alpha diversity analysis was performed using Mothur 1.30.2 and beta diversity analysis using Qiime 1.9.1. Kruskal_Wallis test. Finally, one-way ANOVA was used to detect species that exhibited differences in abundance within each group of microbial communities.

### Statistical methods

The differences between the treatment groups were examined using an unpaired Student’s *t*-test. All data are presented as the mean ± S.E.M. Significant differences between groups were accepted when the *p*-value was less than 0.05. The Spearman’s rank correlation analyses were performed with SPSS 22.0 software (SPSS Inc., IL, United States). Data were presented as mean ± standard deviation (SD). The statistical significance of differences between groups was determined using one-way ANOVA followed by Tukey’s post hoc test or the one-way nonparametric ANOVA Kruskal–Wallis test.

## Results and discussion

### Monosaccharide compositions, infrared spectroscopy and nuclear magnetic resonance analysis of *Dendrobium candidum* polysaccharides

The monosaccharide composition of DCP was analyzed by HPLC, and the monosaccharides in the DCP hydrolysate were identified by comparison with the retention times of the standard monosaccharides (Supplementary Figure S1). We found that the monosaccharide fractions in DCP were mainly composed by Glucose, Mannose, and Glucuronic acid with a molar ratio of 40.12:44.21:0.91.

Infrared spectroscopy (IR) was used to analyze the structure of DCP by using a FT-IR spectrometer (Thermo Scientific Nicolet 6700). As shown in Supplementary Figure S2, three characteristic absorption peaks of polysaccharides were observed at 2900–3700, 1500–1800 and 950–1300 cm^−1^. Two strong absorption bands, each at 3416.4 and 1637.2 cm^−1^ represented the stretching vibration and the bending vibration of O-H groups, respectively. The absorption band at 2925.8 and 1376.97 cm^−1^ were attributed to the stretching vibration and the angular deformation of C-H, respectively. The signal at 1735.3 cm^−1^ belonged to C=O stretching vibration. The absorbance peak at 1248 cm^−1^ was assigned to C-O-C stretching vibration of glycosidic bond. The strong peak appearing at 1027.9 cm^−1^ indicated the existence of α-glycosidic bond. In addition, there was no peak at 1610.5 cm^−1^, that was attributed to the N-H variable angle vibration of -CONH-, revealing that DCP contained relatively little protein.

^1^H and ^13^C NMR spectra were used to further identify the structure of DCP. As shown in Supplementary Figure S3A, the peaks at 5.32 and 5.43 ppm were assigned as α-anomeric proton of mannose and glucose, respectively. β–anomeric proton signal was located near 4.7 ppm, but it was overlapped with the water peak. The protons H-2, H-3, H-4, H-5 and H-6 of both mannose and glucose were present in the range of 3.0–4.2 ppm in the spectrum. However, they were not well separated due to the complicated nature of DCP. Furthermore, the peak near 2.1 ppm was attributed to the proton of acetyl group.

As shown in Supplementary Figure S3B, ^13^C NMR spectra indicated the characteristic anomeric signals at 100.09 and 99.32 ppm for mannose and glucose residues (heterohead carbon). The signal at 76.47 ppm was attributed to C-4, while the signals at 73.32 and 74.99 ppm were attributed to C-5. The peaks at 71.47, 69.97 and 60.44 ppm belonged to C-3, C-2 and C-6, respectively. In addition, the signal near 20 ppm was assigned to CH3 in the acetyl group, and the peak at 172.92 ppm was attributed to carbonyl carbon atom in the acetyl group.

### *Dendrobium candidum* polysaccharides inhibits nitric oxide release and pro-inflammatory cytokine production in LPS-stimulated RAW 264.7 cells

**FIGURE 2:**
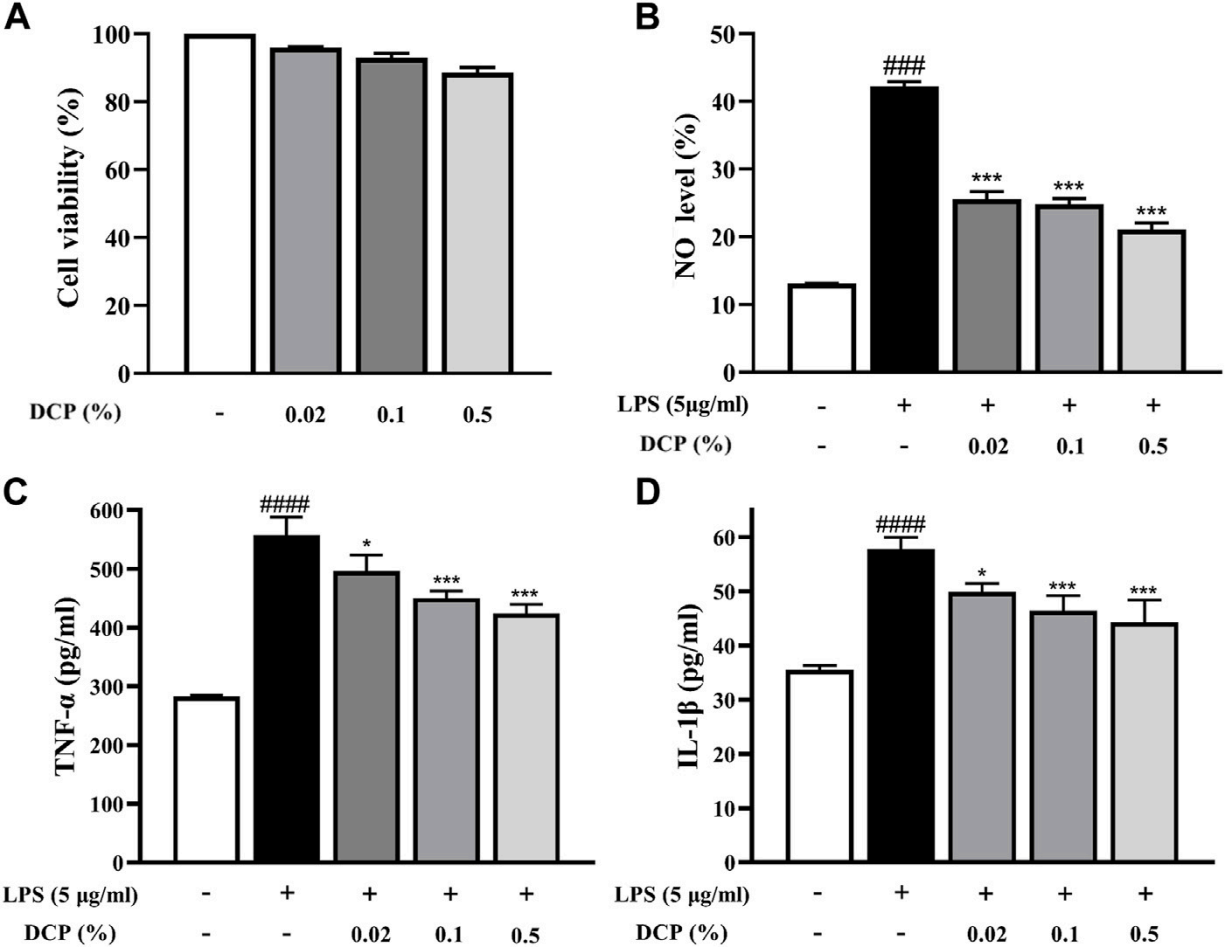
Effects of DCP on RAW 264.7 cell viability **(A)**; Effects of DCP on the LPS-induced NO production **(B)** and proinflammatory cytokine production in RAW 264.7 cells **(C–D)**. RAW 264.7 cells were pre-incubated with 5 μg/ml LPS for 2 h, and then treated with 0.02%, 0.1% and 0.5% DCP for an additional 24 h. Each point represents the mean ± S.E.M. of 3 experiments. ^#^ *p* < 0.05, ^####^ *p* < 0.0001, compared with Control group; *p < 0.05, **p < 0.01, ***p < 0.001 compared with LPS group.

RAW 264.7 cells are a macrophage-like, Abelson leukemia virus-transformed cell line, which is often used to investigate the anti-inflammatory properties of drugs. The effect of DCP on RAW 264.7 cell viability was first determined after incubation for 24 h using MTT assay. As shown in Figure 2A, DCP showed negligible cytotoxicity at a concentration up to a dose of 500 μg/ml.

Macrophages are immune cells involved in the inflammatory response and when inflammation occurs in the body, macrophages secrete pro-inflammatory mediators (including NO) and pro-inflammatory cytokines (such as TNF-α and IL-1β) to promote the inflammatory response (Hou et al., 2020). As can be seen from Figure 2B, the NO secretion level in LPS-stimulated RAW 264.7 cells was obviously higher than that in the Control group (*p* < 0.001). In contrast, DCP significantly reduced the level of NO secretion in LPS-induced RAW 264.7 cells at concentrations of 0.02%, 0.1%, and 0.5% (*p* < 0.001). To understand the inhibitory effects of DCP on inflammation, pro-inflammatory cytokines, IL-1β and TNF-α were investigated in RAW 264.7 cells stimulated by LPS. The expression levels of these pro-inflammatory cytokines showed a significant increase, following stimulation of the cells by LPS (Figures 2C,D). Conversely, treatment with DCP demonstrated a statistically significant decrease of IL-1β and TNF-α, respectively, indicating that DCP had an anti-inflammatory effect s in activated macrophages.

### *Dendrobium candidum* polysaccharides protected BALB/c mice against dinitrofluorobenzene-induced skin damage

**FIGURE 3:**
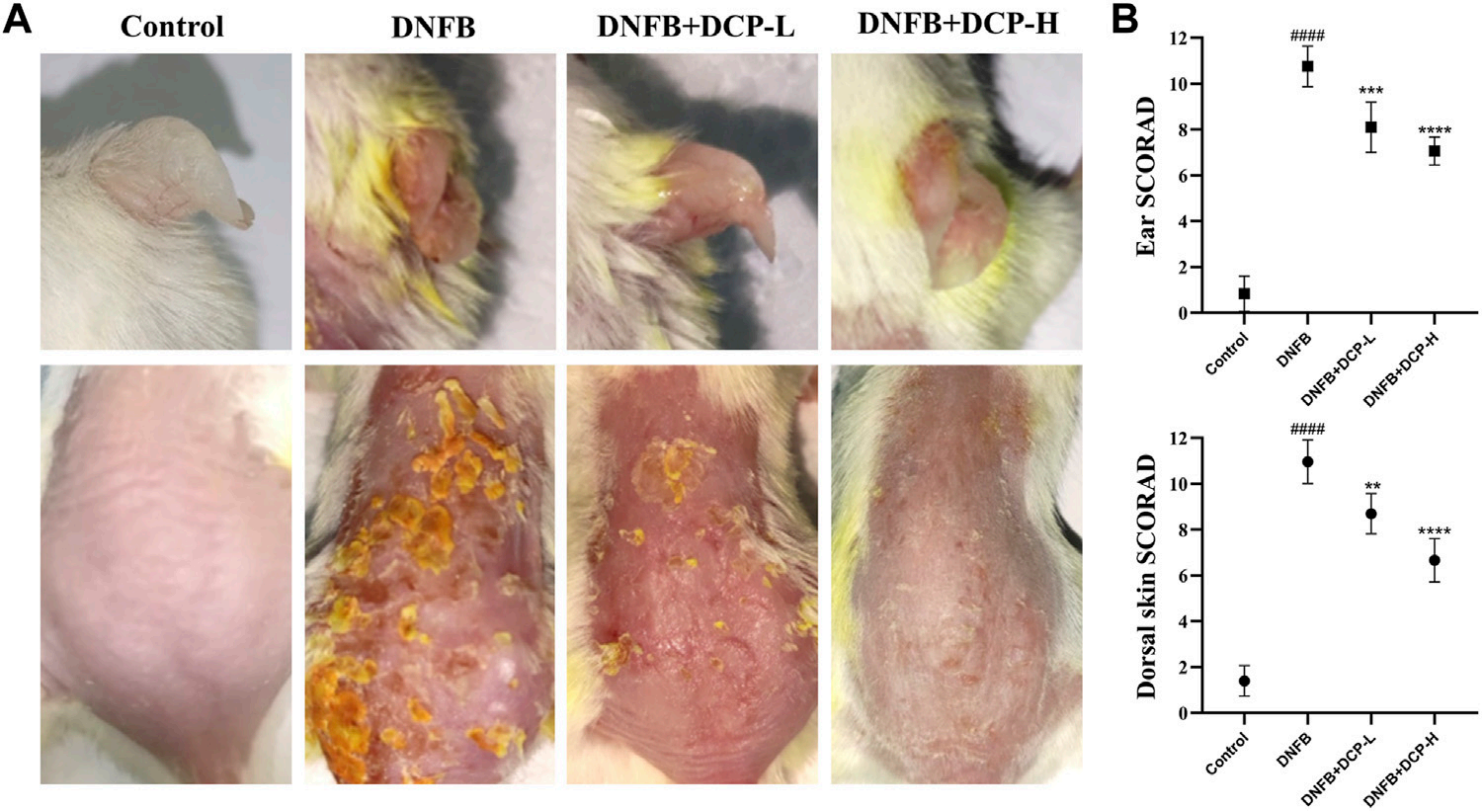
Effects of DCP on the dorsal skin and ear lesions **(A)**, clinical score measurements **(B)**. The dermatitis SCORAD (mean ± SD) for the dorsal skin and ear from each mouse analyzed by *n* = 5 independent investigators in a randomized blinded fashion from 0 (no) to 12 (severe). Each SCORAD represents the mean ± S.E.M. of 5 experiments.

Under specific pathogen-free conditions, repeated subcutaneous treatment with DNFB induces Th2-type skin inflammation in mice similar to that of human AD (Chen et al., 2020), which develop skin lesions, predominantly moderate to severe erythema, hemorrhage, and erosion (Mandlik et al., 2021). To investigate whether DCP can protect BALB/c mice against DNFB-induced skin damage *in vivo*, we first assessed the macroscopic damage of the severity of dorsal and ear skins by using SCORAD, which indicated four symptoms: erythema/hemorrhage, erosion, dryness and scabbing from 0 (no) to 12 (severe). As shown in Figure 3, the dorsal and ear skins were obviously damaged in DNFB-induced mice. However, DCP treatment could clearly improve the severity of debilitating atopic dermatitis lesions. Indeed, the dermatitis score in the DNFB-induced mice was significantly higher for dorsal skin (10.90 ± 0.69) and ear skin (10.76 ± 0.88). Conversely, oral treatment with 50 or 200 mg/kg of DCP markedly suppressed DNFB-induced increases in dermatitis score for dorsal skin (7.30 ± 1.23 or 5.67 ± 1.03) and ear skin (8.10 ± 1.1 or 7.10 ± 0.61).

**FIGURE 4:**
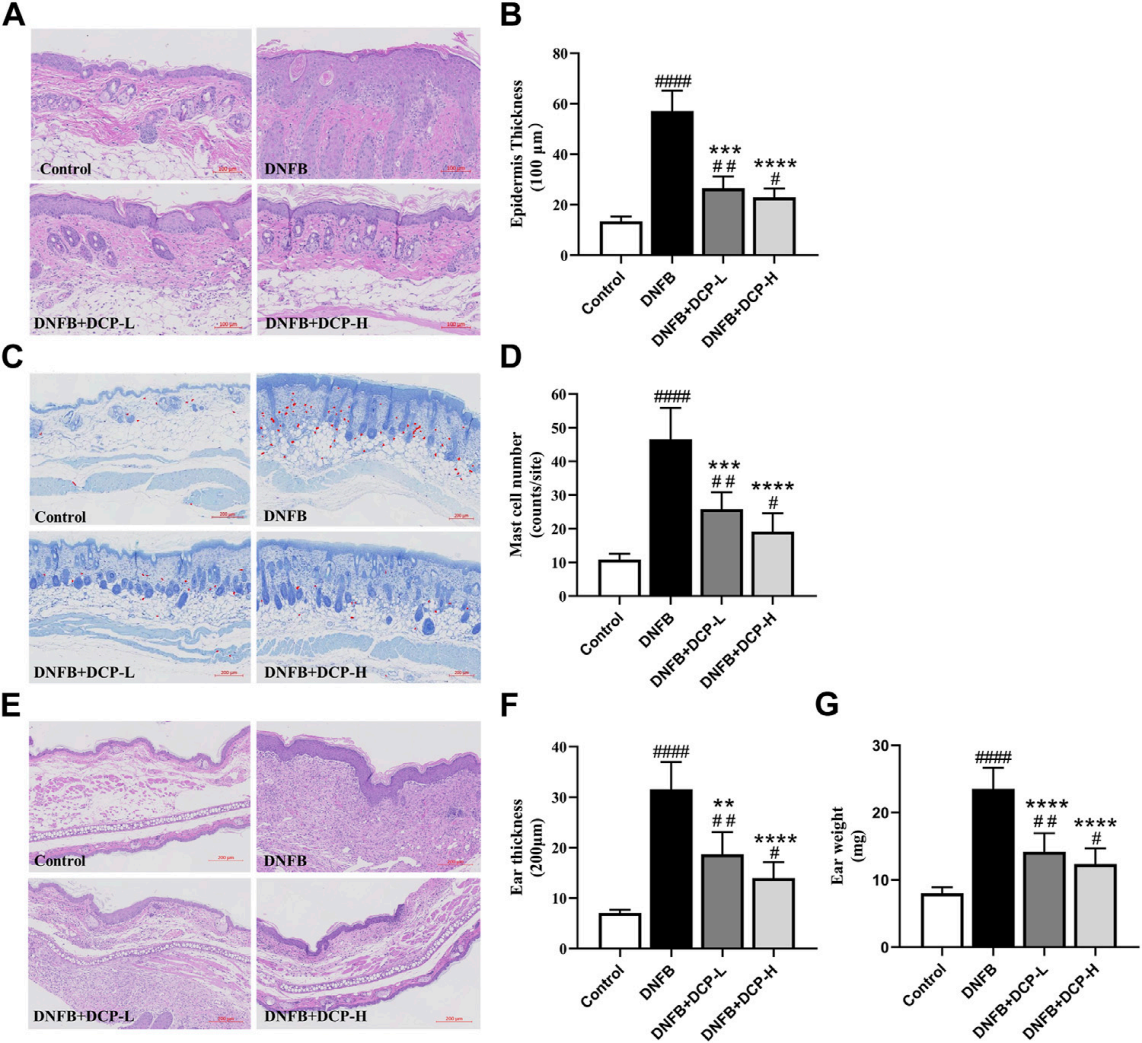
Effect of DCP on AD mice histological features of skin and ear lesions. **(A,E)** The skin lesions were determined by H&E; **(B)** Epidermal thickness was analyzed in H&E-stained tissue (scale bars, 100 μm); **(C,D)** The number of mast cells was analyzed in the TB-stained sections; **(F)** Ear thickness was analyzed in H&E-stained tissue (scale bars, 200 μm), **(G)** and ear weight was measured during the dissection. The values are shown as mean ± S.E.M. (*n* = 6). **p < 0.01, ***p < 0.001 and ****p < 0.0001 vs. the AD model group; ^#^ *p* < 0.05, ^##^ *p* < 0.01 and ^####^ *p* < 0.0001 vs. the Control group.

Then, H&E staining of tissue sections further confirmed significant histological changes in the dorsal and ear skins. As seen in Figures 4A,B,E,F, there were obviously increased epidermal thickness of the dorsal and ear skins in mice which received DNFB stimulation. The mean epidermal thickness was 57.08 ± 8.11 μm for dorsal skin and 31.59 ± 5.36 μm for ear skin, compared to the control group (13.44 ± 1.83 and 7.05 ± 0.65 μm). However, the thickness in the epidermal layer of skin was obviously decreased by treatment of DCP, which was consistent with the ear skin test results. Indeed, DCP treatment reduced the DNFB-induced epidermal thickness of dorsal and ear skins to 26.49 ± 4.75, 18.69 ± 4.38 and 22.89 ± 3.52, 13.97 ± 3.14 μm, respectively. Simultaneously, the ear weight of DNFB-induced mice was also significantly decreased after DCP treatment (from 23.53 ± 3.14 to 14.19 ± 2.74 and 12.37 ± 2.31 mg shown in Figure 4G).

A mast cell, also known as a mastocyte plays a key role in the inflammatory process, and increased mast cells induce skin lesions in the development of AD (Kawakami et al., 2009). The staining of the dorsal skin with toluidine blue, shown in Figures 4C,D, displayed the numbers of mast cells were remarkably increased in the dermal layer of the dorsal skin in DNFB-induced mice, compared with the control. On the contrary, the numbers of mast cells were significantly suppressed after DCP treatment. Histomorphology analysis showed that treatment with DCP could effectively inhibit inflammatory infiltration and attenuate epidermal thickening of the dorsal and ear skins in DNFB-induced mice, which might be attributed to the recovery of DNFB-induced AD-like skin lesions.

### *Dendrobium candidum* polysaccharides modulated gut microbiota in dinitrofluorobenzene-induced atopic dermatitis-like mice

Alterations in the human gut microbiome might have impact on the function of host immune cells, located in the gastrointestinal mucosa and gut-associated lymphoid tissue. The metabolites of these gut microbiome can adjust the immune system balance. The imbalance of gut microbiome (dysbiosis), which triggers the unbalance of the intestinal homeostasis and cause inflammation is a hallmark of several inflammatory diseases, including skin diseases (De Pessemier et al., 2021). Thus, the restoration of the human intestinal microbial dysbiosis has emerged as a promising therapy for inflammatory disorders.

**FIGURE 5:**
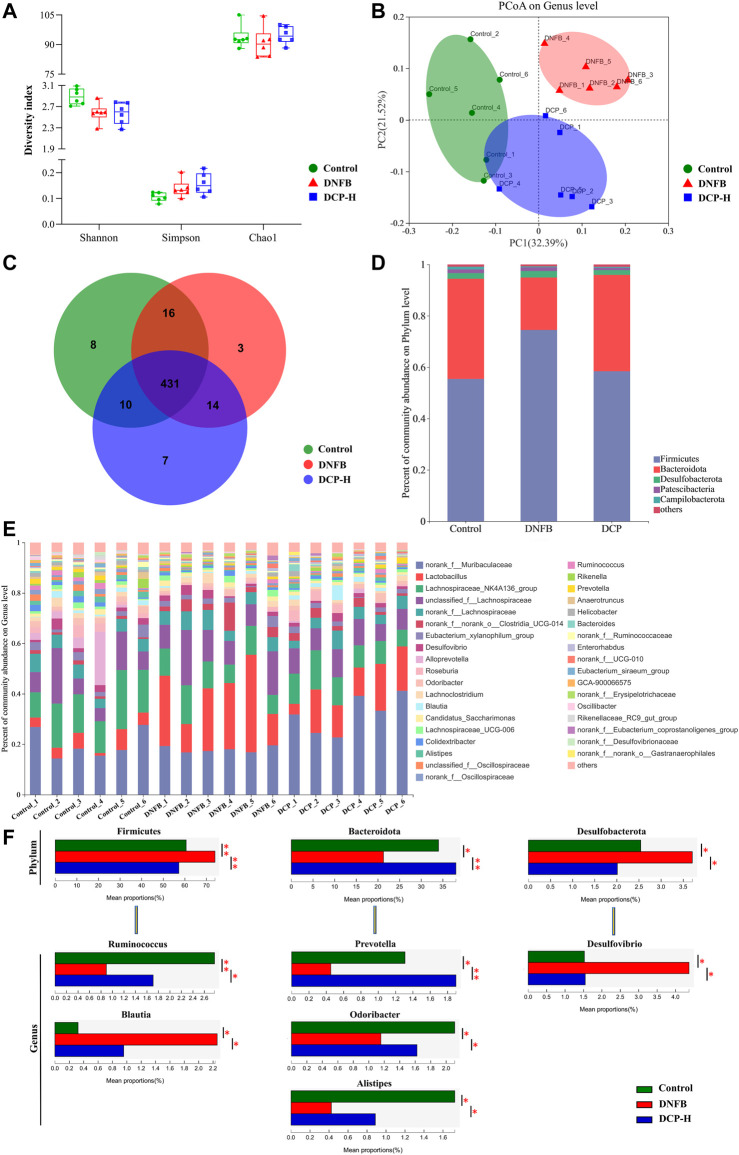
Overview of the gut microbiome in different groups. **(A)** Simpson, Shannon’s and Chao1 indices in α-diversity; **(B)** The principal coordinate analysis (PCoA) of gut microbial on genus level; **(C)** Venn diagram displayed overlapping and shared OTU data among groups; **(D)** A barplot showing the distribution of differences in gut microbial phylum levels; **(E)** The gut microbiome composition at the genus level; **(F)** Relative abundance of the most affected gut microbiota (*p* < 0.05). Data are presented as mean ± SD (*n* = 6). Different * represent statistically significant differences.

In this study, three samples of intestinal contents were randomly selected from each group (Control, DNFB-induced AD-like mice model and 200 mg/kg DCP treatment groups) and microbial 16S rRNA gene sequencing was performed on the Majorb platform. In these three samples, the bacteria belonged to 10 phyla, 15 classes, 34 orders, 52 families, 112 genera, 187 species and 489 operational taxonomic units (OTUs) were identified. In our microbiome analysis, we observed no significant differences in the Simpson, Shannon’s or Chao1 indices (Figure 5A) among three samples, in terms of alpha diversity. To assess the overall structure of the gut microbiota, a PCoA score plot was constructed following β-diversity analysis (Figure 5B). The results showed that the confidence ellipses for the structure of the gut microbiota were separate for the control and DNFB-induced AD-like mouse model groups. However, the confidence interval ellipses for both of DCP treatment and the control groups had a tendency to intersect, indicating that DCP could restore the gut microbial structure of DNFB-induced AD-like mice to a healthy state. Then, a Venn diagram was constructed to check for the presence of OTUs with relative abundance greater than 0.1% in each group (Figure 5C). The majority of OTUs (431 in total) were shared by the three groups. However, a total of 10 OTUs were specifically shared by the control and DCP treatment groups. In addition, a total of 16 OTUs were shared by the control and DNFB-induced AD-like model groups, and 14 OTUs were shared only by the DNFB-induced AD-like model and DCP treatment groups. In total, 3 OTUs were uniquely present in the DNFB-induced AD-like model group, 8 OTUs were in the control group and 7 OTUs were in the DCP treatment group.

Then, the changes in the microbiome population were investigated at phylum level. We found that Firmicutes and Bacteroidetes accounted for more than 95.0% of the total gut microbial composition in all analyzed samples. The proportion of Bacteroidetes and Firmicutes are highly correlated with gut microbiota ecological stability. An increase in Firmicutes/Bacteroidetes (F/B) ratio is implicated in maintaining normal intestinal homeostasis and consequently increase the concentration of short-chain fatty acids (SCFAs) in the intestine (Dei-Cas et al., 2020). SCFAs, as the most abundant microbial metabolites in the colon lumen, serve as a major energy source for epithelial cells and affect the expression of genes required to maintain epithelial barrier and defense functions. They regulate the activity of innate immune cells such as macrophages, neutrophils and dendritic cells, and antigen-specific adaptive immunity mediated by T and B cells (Łoś-Rycharska et al., 2020). Among SCFAs, butyrate is the preferred fuel for the colonic epithelial cells, which stimulate the differentiation of regulatory T cells (Tregs) to maintain the balance between Th1/Th2, thereby exerting anti-inflammatory effects through the decrease of the production of inflammatory factors IFN-γ, IL-4, IL-6 (Tanoue et al., 2016). As shown in Figure 5D, the phyla F/B ratio of the gut microbiota was higher in DNFB-induced mice (3.38 ± 0.56) than in the control group (1.47 ± 0.50). Notably, the treatment of DNFB-induced AD-like mice with DCP promoted the expansion of Bacteroidete populations and the reduction of Firmicute populations, with the F/B ratio of 1.32 ± 0.18, suggesting that DCP could alter the intestinal microbial dysbiosis and bring it closer to the control group.

The relative abundance of bacteria was also analyzed at the genus level (Figures 5E,F). *Alistipes*, *Blautia*, *Desulfovibrio*, *Odoribacter*, *Prevotella*, *Ruminococcus* were the most affected genera in the DCP-treatment group. *Ruminococcus*, belonging to the phylum Firmicutes is a gram-positive anaerobic bacterium, which can ferment polysaccharides into SCFAs, such as acetate, succinate and butylate (Lee et al., 2018a). *Prevotella*, *Alistipes* and *Odoribacter* belong to the phylum *Bacteroidota*. Among them, *Prevotella* are gram-negative anaerobic bacteria, which are one of the main butyrate-producing bacteria in the gut microbiota (Liu et al., 2021). *Alistipes* and *Odoribacter* are known producer of acetate and propionate, which are correlated with inflammatory cytokines in inflammatory bowel disease (IBD) mice. The acetic acid produced by gut microbes is an inhibitor of colitis (Guo et al., 2021). Although there is no direct evidence that acetate and propionate are directly associated with AD, this kind of SCFAs have biological activity to suppress inflammation and play an important role in gut health (Tedelind et al., 2007). In this study, we noted that DNFB-induced AD-like mice reduced these SCFAs-producing bacteria, compared with the healthy control group. However, they were obviously increased in the DCP-treated group and the relative abundance of these intestinal bacteria was closer to the control group, indicating that the gut microbiome-derived metabolites SCFAs are implicated in the attenuation of inflammation of DNFB-induced AD-like mice. Both of *Desulfovibrio* and *Blautia* are obligate anaerobic intestinal bacteria. *Desulfovibrio* is a genus of gram-negative sulfate-reducing bacteria, which are capable to reduce sulfate to sulfide and then to hydrogen sulfide (H2S). Endogenous H2S is the third gaseous signal molecule with biological roles, which affects intestinal epithelial cells and causes a variety of the host intestinal diseases (Verstreken et al., 2012). Several clinical studies demonstrated that increased *Desulfovibrio* might be manifested as an important feature of acute and chronic ulcerative colitis (Liu et al., 2020). Liu et al. (2020) also reported that AD patients exhibited notable enrichments of *Desulfovibrio* in the human gut microbiome. *Blautia* is a new functional genus with probiotic characteristics, which is significantly associated with host dysfunction, including cancer and various inflammatory diseases, but the direct causal relationship between diseases and abundance is not yet clear. However, the abundance of *Blautia* is found to be higher in patients with AD than in healthy control (Ye et al., 2021). In this study, the relative abundance of *Desulfovibrio* and *Blautia* was significantly higher in DNFB-induced AD-like mice, compared with the health mice. However, DCP treatment could restore the relative abundance of these gut bacteria, which was much closer to that of the health mice group.

### Effect of *Dendrobium candidum* polysaccharides on total serum immunoglobulin E levels and inflammatory cytokines in dorsal skin of DNCB-induced atopic dermatitis-like mice

**FIGURE 6:**
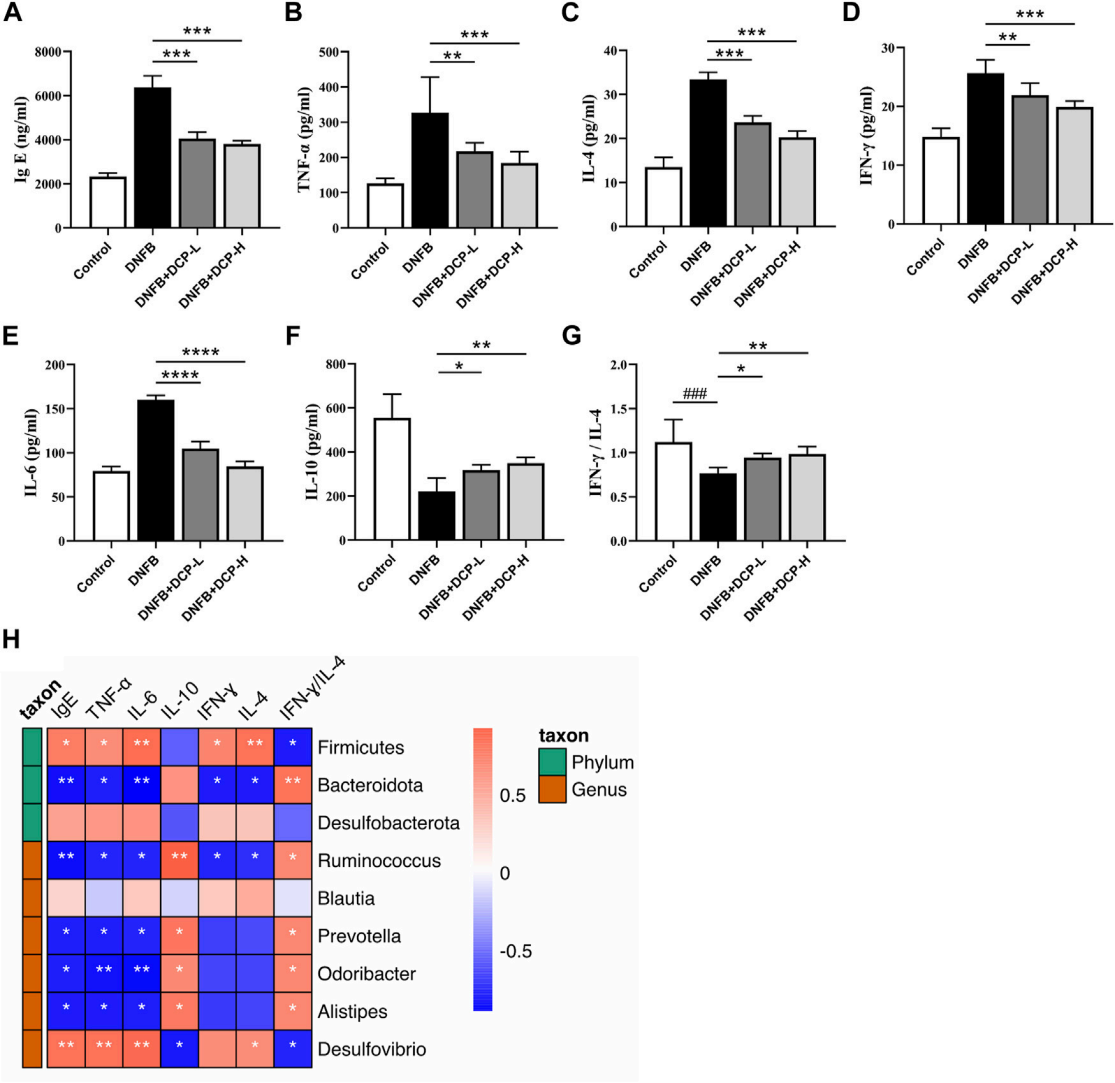
Effect of DCP on inflammatory cytokines and total serum IgE and its correlation with gut bacterial genera. **(A)** Serum concentrations of IgE; **(B–F)** Quantitative analysis of inflammatory cytokines in mouse dorsal skin; **(G)** The ratio of IFN-γ/IL-4; **(H)** Heatmap of correlation between level of cytokines and relative abundance of gut bacterial genera. Each point represents the mean ± S.E.M. of six experiments. **p < 0.01, ***p < 0.001 and ****p < 0.0001 vs. the DNFB (AD model) group. Red indicates positive correlation; blue indicates negative correlation. *p < 0.05, **p < 0.01.

Immunoglobulin E (IgE) are implicated in the pathogenesis of AD, which can activate mast cells through FcεRI, triggering immediate mast cell degranulation and release of inflammatory mediators, including those promoting Th2-polarized T cell responses. The upregulation of serum total IgE is an important criterion for measuring specific dermatitis (Weidinger and Novak, 2016) and anti-IgE therapy was also found to be effective in the treatment of AD (Liu et al., 2011). Indeed, an elevated serum total IgE level was confirmed in the DNFB-induced AD-like murine model (Figure 6A), compared with the control group (*p* < 0.001). However, serum IgE levels were effectively reduced in DNFB-induced AD-like mice after 2-week treatment of DCP (*p* < 0.001) in a dose-dependent manner, further confirming DCP could ameliorate skin damage and inflammatory responses.

Gut microbial species and genera are closely linked to the responses of inflammatory cytokines and there is a strong impact of microbial metabolic metabolites on cytokine production (Schirmer et al., 2016). The dysbiotic gut community also alters the Th1/Th2 balance in favor of a Th2 response, thereby resulting in the abnormal production of inflammatory cytokines. The ratio of Th1-associated IFN-γ and Th2-associated IL-4 is often used as an indicator of Th1/Th2 balance (Liu et al., 2014). It is also known that an anti-inflammatory cytokine IL-10 is closely associated with regulatory T cells (Tregs), which migrate from peripheral and inflamed tissues to draining lymph nodes and suppress a variety of pathological immune responses (Kowalska-Olędzka et al., 2019). As shown in Figure 6, we evaluated the inhibitory effects of DCP on Th1/Th2-related cytokines (TNF-α, IFN-γ, IL-4 and IL-6) and anti-inflammatory cytokine IL-10 in dorsal skin tissue, and noted that the levels of TNF-α, IFN-γ, IL-4 and IL-6 were significantly upregulated and the level of IL-10 was down-regulated in the dorsal skin of DNFB-induced AD-like mice, whereas, these protein levels were obviously modified by DCP in a dose-dependent manner (Figures 6B–F) and the ratio of IFN-γ/IL-4 was significantly increased, which was closer to that of healthy controls (Figure 6G).

To further clarify the correlation between the changes of gut microbiota and the levels of inflammatory cytokines and IgE as well as IFN-γ/IL-4 ratio, we calculated the correlation using Spearman’s analysis method. As shown in Figure 6H, the abundance of *Desulfovibrio* belonging to the phylum *Desulfobacterota* was significantly positively correlated with the pro-inflammatory factors TNF-α and IL-6 (*p* < 0.01), and negatively correlated with the anti-inflammatory cytokine IL-10 (*p* < 0.05). It was also observed that the beneficial bacteria including *Alistipes*, *Odoribacter*, *Prevotella* and *Ruminococcus* were negatively correlated with TNF-α, IL-6, and IFN-γ, and positively correlated with IL-10 (*p* < 0.05). Moreover, at the genus level, *Ruminococcus* belonging to the phylum Firmicutes and *Alistipes*, *Odoribacter*, and *Prevotella* belonging to the phylum *Bacteroidota* were positively correlated with the ratio of IFN-γ/IL-4 (*p* < 0.05) and negatively correlated with total serum IgE levels (*p* < 0.05). These results showed that gut microbial variations were highly correlated with the aberrant expression of pro- and anti-inflammatory cytokines, which could upregulate the level of IgE in serum of AD mice.

## Conclusion

In summary, our results demonstrated the anti-atopic dermatitis effects of DCP in DNFB-induced AD-like mice and provided evidence for the modulatory effects of DCP on the gut microbiota through the restoration of the rebalancing of the ecology of the gut bacterial community and the rebalance of probiotics and pathobionts. DCP treatment could not only decrease IgE content in the serum, which was implicated in the pathogenesis of AD, but also modify the expression of several typical pro- and anti-inflammatory cytokines. Spearman’s correlation analysis further corroborated that the intestinal microbial alterations was closely linked to the expression of inflammatory cytokines, which might be attributed to the recovery of DNFB-induced AD-like skin lesions after treatment with DCP leading us to propose DCP as a promising novel microbiota-modulating agent for the treatment of AD.

## Data Availability

The datasets presented in this study can be found in online repositories. The names of the repository/repositories and accession number(s) can be found below: NCBI BioProject with accession number: PRJNA868518, https://www.ncbi.nlm.nih.gov/bioproject/PRJNA868518.
