# Supplementary material for: Dendrobium candidum polysaccharide reduce atopic dermatitis symptoms and modulate gut microbiota in DNFB-induced AD-like mice
Source: Front Physiol. 2022 Sep 9;13:976421. doi: 10.3389/fphys.2022.976421 (PMC9500176; doi:10.3389/fphys.2022.976421)
Supplement: Supplementary file 1 [file DataSheet1.docx]

Supplementary Material

# DCP preparation and component analysis

In this experiment, DC and distilled water (approximately 20 times the weight of DC) were added to a round bottom flask and boiled for 2 hours and filtered. Distilled water (approximately 20 times the weight of DC) was added again to the round bottom flask and boiled for 2 hours and filtered. The extracts were then combined and concentrated to a viscous state. The round bottom flask was precipitated by adding 80% ethanol, filtered, lyophilized, and stored at 4 ℃. The DCP was dissolved in phosphate-buffered saline (PBS) and then filtered through a 0.22 mm syringe filter and stored.

# Analysis of monosaccharide composition

This experiment weighs 2 mg DCP and the standard of rhamnose, L-Arabinose, Galactose, glucose, Xylose, Mannose, ribose, D-Galacturonic acid, Glucuronic acid. And then, 1 mL of 2 mol / L trifluoroacetic acid was added to the sample and hydrolyzed in oil at 110 ℃ for 3 hours. After the sample is cooled, the trifluoroacetic acid remaining in the sample is blown off with nitrogen and a small amount of methanol is added, and finally dried with nitrogen. Repeat 3 times to remove all trifluoroacetic acid. Finally, the sample was configured as a 40 ppm standard solution. The sample was tested with HPLC (chromatographic column: TSK gel G4000 PWxl, 7.8 mm × 300 mm; concentration of C-AEP-1-2: 1 mg/mL; sample size: 20 μL; column temperature: 30℃; detector: refractive index detector, detecting temperature 35 °C, mobile phase: water).

# Analysis of infrared spectroscopy and nuclear magnetic resonance

In infrared spectroscopy, the mixture of DCP (1 mg) and the highly pure potassium bromide (150 mg) was pressed into tableting as the infrared absorption spectrum. The scanning range is 400 cm-1-4000 cm-1, the resolution ratio is 4 cm-1 AND the scanning times are 16.

In nuclear magnetic resonance, 40 mg DCP was dissolved in 0.6 mL Deuterium oxide (D2O). The information of 1H-NMR and 13C-NMR were collected with NMR spectrometer. (AVIII-400M, Bruker, Switzerland).


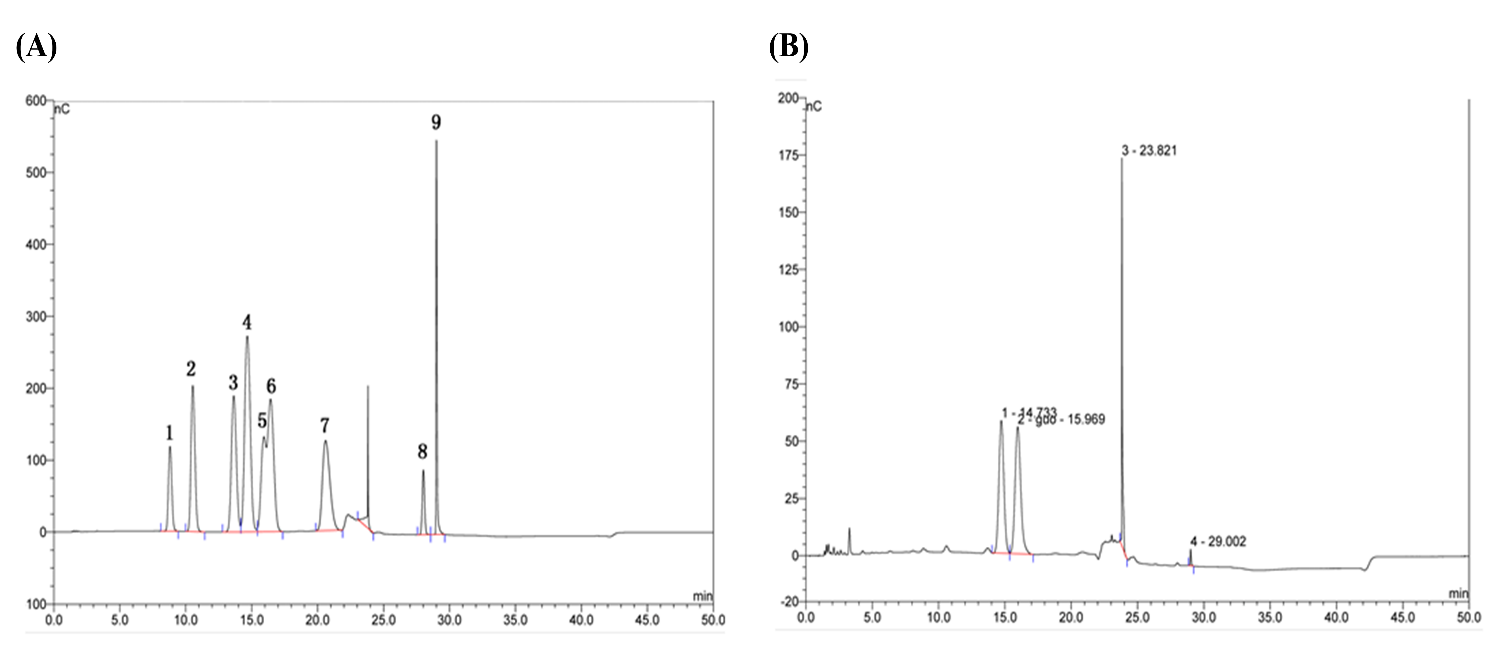


**Figure S1.** HPLC spectra of standards **(A)** and DCP **(B)**. 1-Rhamnose, 2-L-Arabinose, 3-Galactose, 4-Glucose, 5-Xylose, 6-Mannose, 7-Ribose, 8-D-Galacturonic acid and 9-Glucuronic acid in **(A)**.


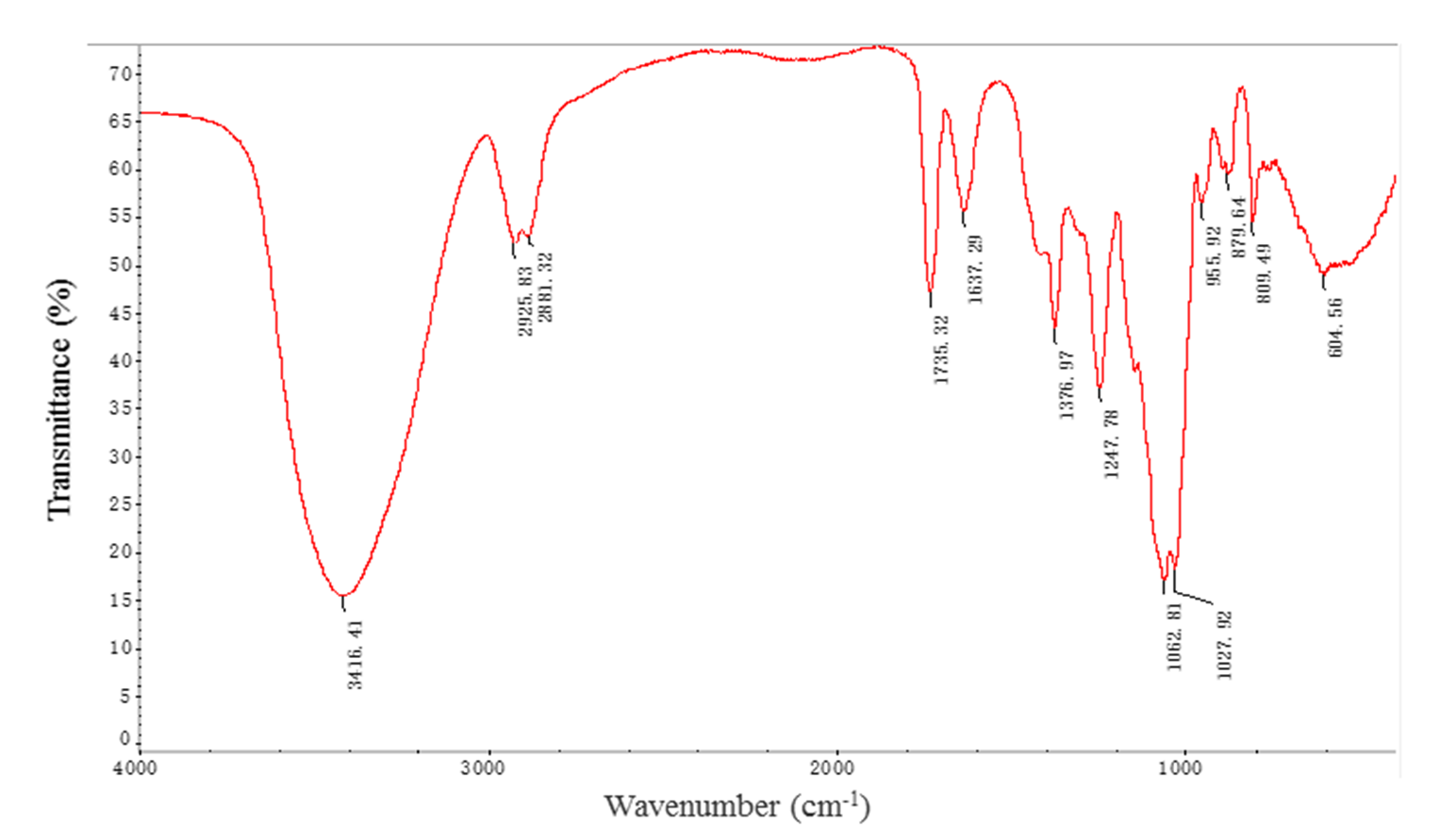


**Figure S2.** Infrared spectrum of DCP.


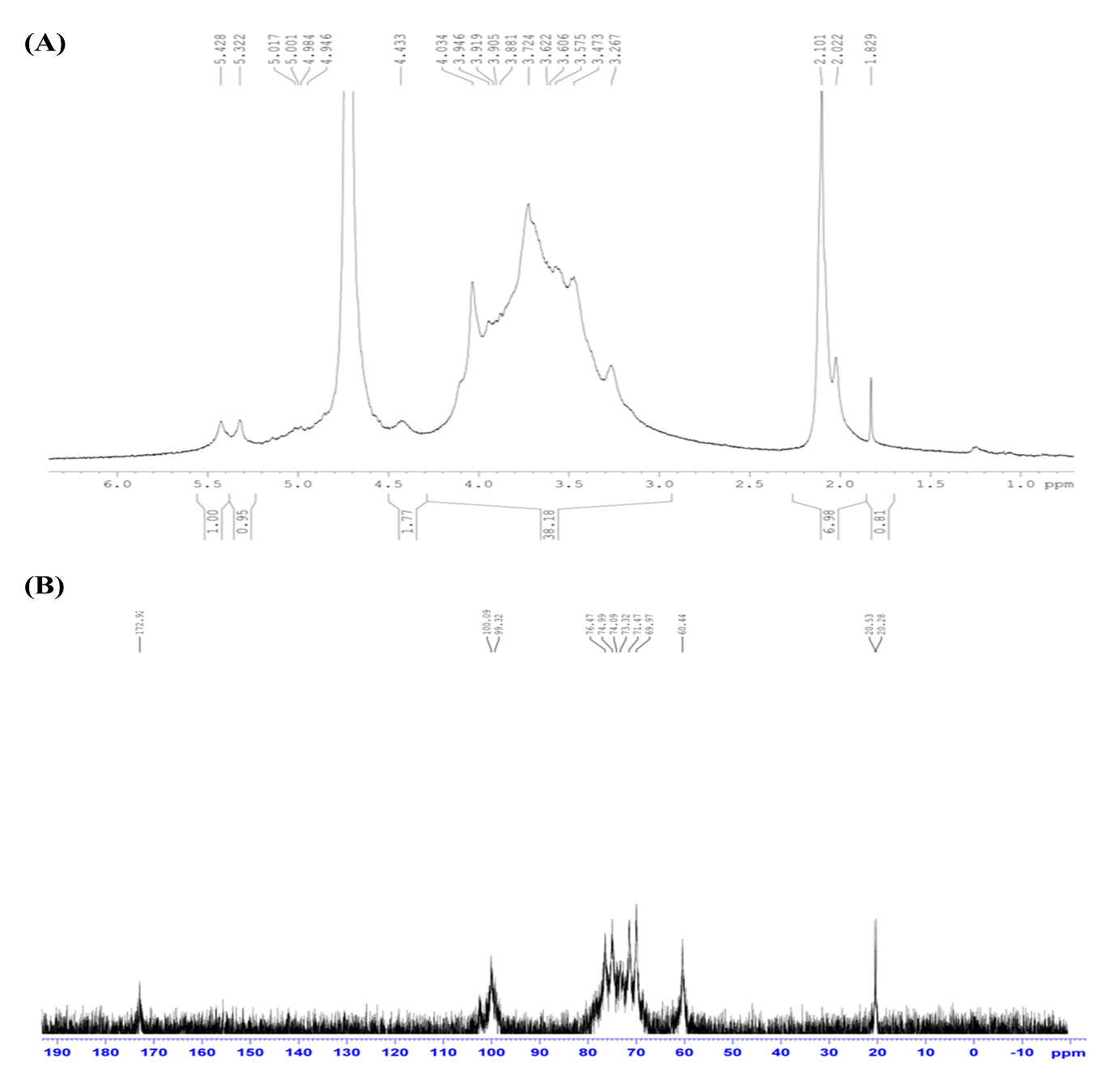


**Figure S3.** ^1^H NMR **(A)** and ^13^C NMR **(B)** of DCP.
